# Infantile Cataracts Associated with a Homozygous Missense *MSMO1* Variant—Case Report and Literature Review

**DOI:** 10.3390/reports9010045

**Published:** 2026-01-30

**Authors:** Nick Hassas, Andy Drackley, Jelena Ivanisevic, Hantamalala Ralay Ranaivo, Sudhi P. Kurup

**Affiliations:** 1 Division of Ophthalmology, Ann & Robert H. Lurie Children’s Hospital of Chicago, Chicago, IL 60611, USA; nick.hassas@my.rfums.org (N.H.); hralay@luriechildrens.org (H.R.R.); 2 Edwards Family Division of Genetics & Rare Diseases, Ann & Robert H. Lurie Children’s Hospital of Chicago, Chicago, IL 60611, USA; adrackley@luriechildrens.org (A.D.); jivanisevic@luriechildrens.org (J.I.); 3 Department of Pediatrics, Feinberg School of Medicine, Northwestern University, Chicago, IL 60611, USA; 4 Department of Ophthalmology, Feinberg School of Medicine, Northwestern University, Chicago, IL 60611, USA

**Keywords:** *MSMO1*, infantile cataracts, variant of uncertain significance, next-generation sequencing, cholesterol synthesis

## Abstract

**Background and Clinical Significance**: *MSMO1*, encoding a key enzyme in the cholesterol synthesis pathway, is associated with an autosomal recessive condition characterized by microcephaly, ocular abnormalities, growth delay, psoriasiform dermatitis, immune dysfunction, and intellectual disability. **Case Presentation:** This report describes a patient presenting with global developmental delay and bilateral infantile cataracts found to harbor a homozygous likely pathogenic *MSMO1* variant and reviews the literature on MSMO1 deficiency and its association with infantile cataracts. **Conclusions**: The mechanism of early lens opacification is thought to result from impaired cholesterol synthesis, altering the lipid composition of the lens membrane and leading to early cataract formation. This case expands our understanding of MSMO1 deficiency and highlights the critical role of cholesterol biosynthesis in early lens development.

## 1. Introduction and Clinical Significance

Infantile cataracts, defined as any opacity of the crystalline lens present within the first year of life, are most often caused by a variety of genetic abnormalities that alter normal lens homeostasis [1]. Emerging evidence suggests that disruptions in the cholesterol synthesis pathway, an essential component of the lens’s cholesterol-rich membranes, represents a mechanism of early cataract formation [2]. This relationship is exemplified by *MSMO1*, a gene that encodes for sterol-C4-methyl oxidase, which is an enzyme in the sterol-C4-demethylation step of cholesterol synthesis. Thus, its deficiency leads to the accumulation of methylsterol intermediates and reduced cholesterol availability within the lens, disrupting the lens membrane and leading to early cataract formation [3].

We present a patient with bilateral infantile cataracts and developmental delay who was found to harbor a homozygous *MSMO1* variant that was re-classified as likely pathogenic by our study team and describe it in the context of a brief review of the literature on MSMO1 deficiency and its likely association with infantile cataracts.

## 2. Case Presentation

### 2.1. Patient Information and History

Our patient is a Hispanic male who initially presented at 5 years old for visually significant, bilateral infantile cataracts and alternating esotropia. Per parental report, he had been diagnosed with cataracts at 8 months of age in Mexico, but there had been no interim eye exams or interventions since then.

**Figure 1:**
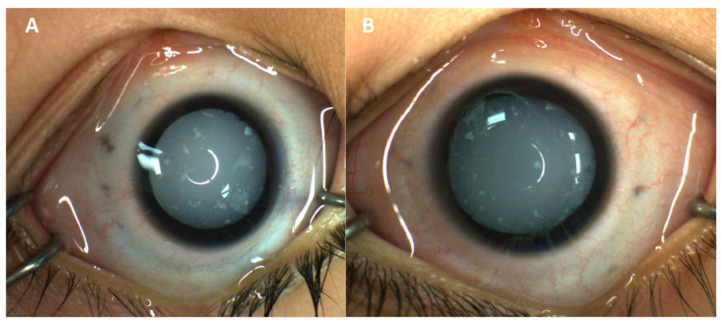
External photography of right (**A**) and left (**B**) eyes, highlighting diffuse cataracts bilaterally with scattered, white fleck-like opacities in the anterior lens after pharmacologic dilation.

Eye exam at presentation showed blink-to-light response in each eye with a moderately large esotropia. Intraocular pressures were within normal limits. The external exam was normal. Anterior segments were notable for diffuse cataracts bilaterally with scattered, white fleck-like opacities in the anterior lens (Figure 1). Posterior segments could not be visualized due to media clarity; however, B-scan ultrasonography was unremarkable. Eye exam under anesthesia revealed increased axial length on A-scan ultrasonography (23.35 mm in the right eye, 24.32 mm in the left eye). The patient underwent lensectomy and anterior vitrectomy with intraocular lens implantation; of note, the cataracts had an intumescent appearance with significant lenticular liquefaction. After cataract surgery, the posterior segment was also found to be unremarkable on exam. The patient still only showed perception to light, but parents felt there was some improvement in this regard.

He was additionally evaluated by Neurology due to his developmental delays; upon evaluation, low axial tone with increased appendicular tone and microcephaly (head circumference: 46.4 cm, Z ≤ 2.05) were confirmed, prompting neuroimaging of the brain and spine. Non-contrast MRI of the brain revealed diffuse reduction in white matter volume in the cerebral hemispheres, with foci of T2 FLAIR hyperintensity in the posterior white matter, which were associated with mild prominence of the lateral and third ventricles, undulating margins of the lateral ventricles, and thinning of the corpus callosum. Non-contrast MRI of the entire spine revealed no abnormalities. The patient has not undergone formal dermatological evaluation by a dermatologist to date. Upon physical examination, the skin demonstrated segmental confluent hyperpigmentation of his right trunk without axillary or inguinal freckling, with the patient’s mother reporting that he had no history of psoriasis or dry skin. The patient’s fasting serum lipid measurements, including total cholesterol, triglycerides, HDL cholesterol, and LDL cholesterol, were all within normal limits. Our patient is non-verbal and non-ambulatory with a diagnosis of spastic quadriplegia.

Family history was notable for parental consanguinity, with the patient’s parents being second cousins. Immediate family members revealed good general health, but maternal and paternal first cousins were reported as having poor vision and developmental delays. The patient’s birth and prenatal history were without complications, noting negative TORCH testing and normal fetal anatomy ultrasound.

### 2.2. Genetic Testing

Our proband completed whole exome sequencing (WES) and mitochondrial genome analyses at a reference laboratory. The analyses were completed with the proband and maternal samples only.

## 3. Discussion

We report a six-year-old male with bilateral infantile cataracts, global developmental delay, and homozygous variants in *MSMO1* and *PLEKHG2* identified by exome sequencing. WES identified two homozygous variants both classified and reported by the reference laboratory as variants of uncertain significance (VUS): NM_006745.5(MSMO1):c.343G>A (p.Gly115Arg) and NM_022835.3(PLEKHG2):c.3964dup (p.Gln1322Profs*19). *MSMO1* c.343G>A (p.Gly115Arg) has been reported previously as homozygous in a two-year-old individual with congenital cataracts, microcephaly, developmental and growth delay, and joint contractures, with serum cholesterol levels around 90 mg/dL [4]; at five years old, that individual was noted to have dry skin but without history of psoriasiform rash.

*MSMO1* c.343G>A itself is rare in the population, with just two heterozygous individuals in gnomAD v4.1. The amino acid substitution is strongly predicted by AlphaMissense [5] to be damaging to protein function (score = 0.9933). It is in gnomAD v4.1 in just two heterozygous individuals and has been previously identified in the homozygous state in an individual with an overlapping clinical phenotype [4]. Based on its computational predictions, rarity in the population, and presence in the homozygous state in multiple individuals with suspected MSMO1 deficiency, re-assessment of the variant’s clinical significance by the study team was consistent with a classification as likely pathogenic (ACMG-AMP criteria applied: PP3_Strong, PM3, and PM2_Supporting) [6].

Sterol profiles in serum and skin scales from the individual in He et al. [4] with homozygous *MSMO1* c.343G>A were abnormal, with significantly elevated 4α-monomethyl sterols and 4, 4′-dimethyl sterols, with similar profiles seen in their other patients with MSMO1 deficiency and biallelic *MSMO1* variants. Neither 4-carboxylmethylsterol nor 4-methylsterone was detected in skin scales or skin fibroblasts from these patients [4]. These findings are consistent with a defect at the initial step of sterol-C4-demethylation. As He et al. noted, these individuals have marked elevation of both 4α-monomethyl sterols and 4, 4′-dimethyl sterols, suggesting they do retain some residual enzyme activity. Interestingly, despite diminishment in this enzyme activity apparently sufficient to cause disease, to our knowledge, no patients have yet been reported with predicted loss of function variants (e.g., nonsense, frameshift) or with a demonstrated complete loss of sterol-C4-methyl oxidase activity.

Previous publications have described several patients with biallelic *MSMO1* variants with psoriasiform dermatitis [3,4,7], in addition to infantile cataracts and development delays. However, our patient has not displayed any psoriasis skin manifestations thus far, only noting the segmental confluent hyperpigmentation without axillary or inguinal freckling. Notably similar to our patient, neither the female individual diagnosed at 2 years old [4] nor the male diagnosed at 8 months old [8] displayed psoriasiform dermatitis at the time of publication. This may suggest that certain *MSMO1* variants primarily affect cholesterol homeostasis within the lens, leading to isolated ocular manifestations without cutaneous abnormalities. As the compound heterozygous variants (p.Gly202Glu, p.Tyr244Cys) identified in the patient reported in Frisso et al. [8] are both in the region of *MSMO1* encoding the enzyme’s fatty acid hydroxylase domain [9], while c.343G>A (p.Gly115Arg) is not, the explanation for different variants leading to more or less pleiotropy is likely more complex than simply based on the region of the gene in which the variants occur. This case adds to the limited number of reports suggesting MSMO1 deficiency can lead to early lens opacification without additional systemic features.

The patient’s *PLEKHG2* single base pair duplication introduces a frameshift and resultant premature stop codon in the last exon of the gene; it is not expected to trigger degradation through nonsense-mediated decay and is instead predicted to result in a slightly truncated protein. The limited functional and clinical data available on the importance of the protein’s C-terminus make this variant’s impact unclear. *PLEKHG2* is involved in cell proliferation and migration, with variation in it thought to be associated with leukodystrophy. Our patient’s MRI findings suggest remote white matter injury rather than the leukodystrophy presentation seen in *PLEKHG2*-related disease, with the patient’s head circumference suggesting primary rather than secondary/acquired microcephaly associated with *PLEKHG2* [7,10,11,12,13]; it is therefore unlikely that this homozygous VUS is clinically relevant to the present patient.

Expanding the number of reports of individuals with confirmed or presumed *MSMO1*-related disease is critical for clinicians attempting to correlate genetic testing results clinically, as well as for laboratories in their interpretation, classification, and reporting of identified variants. Greater recognition of these *MSMO1*-related cataracts further highlights the critical role of cholesterol in lens development and may lead to new avenues for investigating metabolic contributions to cataracts.

## 4. Conclusions

We describe a six-year-old male with bilateral infantile cataracts and developmental delays likely caused by a homozygous *MSMO1* variant, underscoring the importance of cholesterol synthesis in the lens. This case contributes to the growing number of reports of MSMO1-associated cataracts without the skin manifestations described in the prior reports and reinforces the importance of further characterization of this gene–disease relationship to improve clinical and molecular diagnoses.

## Data Availability

The original data presented in this study are available on reasonable request from the corresponding author. The data are not publicly available due to privacy concerns.
